# Peripheral serum copper metabolite levels measured by ultrafiltration aid in the clinical screening of Alzheimer’s disease

**DOI:** 10.3389/fpsyt.2026.1954419

**Published:** 2026-10-14

**Authors:** Yi Zhang, Longhui Li, Houxian Ouyang, Zheng Zhao, Xi Mei, Conglong Qiu

**Affiliations:** Department of Psychiatry, Zhejiang Key Laboratory of Drug Addiction & Brain Health, Affiliated Kangning Hospital of Ningbo University (Ningbo Kangning Hospital), Ningbo, Zhejiang, China

**Keywords:** Alzheimer’s disease, peripheral blood, precise diagnosis, serum copper, ultrafiltration

## Abstract

**Background:**

The number of patients with Alzheimer’s disease (AD) is increasing, yet misdiagnosis remains common and early accurate diagnosis is lacking. Previous studies have suggested abnormalities in peripheral serum copper levels in AD patients. This study sought to explore the diagnostic value of peripheral serum copper-related biomarkers for clinically diagnosed AD.

**Methods:**

Peripheral serum samples were collected from 115 clinically diagnosed AD patients and 96 healthy controls (HC). A preliminary experiment on five samples determined the use of a 10 kDa ultrafiltration membrane. Four serum copper parameters—serum total copper (tot-Cu), free copper (free-Cu), serum immunological ceruloplasmin concentration (iCp), and ceruloplasmin enzymatic activity (eCp)—were quantitatively measured in all samples using ultrafiltration, atomic absorption spectrometry, and ELISA.

**Results:**

Peripheral tot-Cu and free-Cu levels in clinically diagnosed AD patients were significantly elevated in the early stage of the disease, while iCp, eCp, and the iCp/eCp ratio were significantly decreased. The diagnostic efficacy of tot-Cu and free-Cu for these Alzheimer’s disease patients was significantly higher than that of the Mini-Mental State Examination (MMSE) score. Nevertheless, these indicators could not distinguish different severity stages of clinically phenotyped AD.

**Conclusion:**

Our results indicate that tot-Cu or free-Cu holds promise as biomarkers for the clinical screening of AD; peripheral copper metabolic dysregulation may be involved in the pathogenesis of clinically phenotyped AD.

## Introduction

Alzheimer’s disease (AD) is a slowly progressive neurodegenerative disorder clinically characterized by global cognitive impairment, progressive decline in activities of daily living, and neuropsychiatric symptoms (1). AD leads to disability, loss of speech, and even immobility (2), severe cases can be fatal (3), and it imposes a heavy economic burden (4). Increasing age greatly increases the prevalence of AD. Affected by the aging population in China, the number of people with AD has exceeded 10 million (5).

Currently, the diagnosis of AD is often based on clinical symptom criteria, such as the International Classification of Diseases (ICD-11) (6), the Diagnostic and Statistical Manual of Mental Disorders, Fifth Edition (DSM-V) (7), and the National Institute on Aging-Alzheimer’s Association (NIA-AA) 2011 criteria (8). Clinical diagnoses made according to these criteria are susceptible to various subjective factors, such as clinical experience, leading to variable accuracy. An autopsy study showed that among patients diagnosed with AD during life, more than 50% had pathological findings of dementia with Lewy bodies (DLB) or other types of dementia (9). However, experienced clinicians using diagnostic criteria combined with neuropsychological tests and neuroimaging can achieve diagnostic accuracy as high as 81.6% (10). Biological diagnostic methods for AD (11, 12) are more objective and can shift the diagnostic window forward by 10–20 years (11), facilitating early detection of dementia. However, due to equipment requirements and poor patient compliance, they are difficult to implement on a large scale.

In clinical practice, most patients seek medical attention only after obvious cognitive decline occurs, when Alzheimer’s disease is clinically diagnosed (13), often missing the optimal treatment window. Early effective treatment of AD can significantly improve the prognosis (14). New drugs, such as disease-modifying therapies (e.g., lecanemab, donanemab), are only effective for mild cognitive impairment (MCI) or mild AD (15, 16). Therefore, improving the detection rate at the early stage of the disease is crucial. There is a need for an objective, easy-to-implement, low-cost, high-sensitivity diagnostic method that can show positivity in the early stages of AD.

Studies have shown that copper metabolites in human serum are associated with AD (17–20). Copper in the human body is mainly obtained from food intake, and the sum of all copper in serum is serum total copper (tot-Cu). 85%–95% of ingested copper binds to serum ceruloplasmin (Cp) and is stored in the liver (21). 0.5%–5% of serum copper is free in the peripheral blood (18) as free copper (free-Cu). Free-Cu can freely cross the blood-brain barrier (BBB) and is involved in oxidative damage in the central nervous system (CNS) (22), increasing the neurotoxicity of Aβ (23, 24). Cp is closely related to the ATP7B protein, and mutations in the ATP7B gene are associated with AD risk (25). Serum immunological ceruloplasmin concentration (iCp) and ceruloplasmin enzyme activity (eCp) are involved in human copper metabolism. Some studies have shown that the eCp/iCp ratio is decreased in AD populations (20).

Previous studies on human serum copper metabolites have mostly been limited to one or two metabolites and have rarely associated them with AD severity. This study aims to comprehensively characterize serum copper-metabolite levels in patients with clinically diagnosed AD across different severity stages, and to evaluate the diagnostic performance of these metabolites. For detection of the key metabolite free-Cu, we used ultrafiltration instead of traditional methods, making the results more reliable and objective. The results of this study demonstrate that human peripheral serum copper-metabolite levels can effectively discriminate patients with clinically phenotyped AD from healthy controls. Their diagnostic performance is superior to that of the Mini-Mental State Examination (MMSE). Moreover, these metabolites exhibit favorable detection capacity even in the mild disease stage within our clinically-diagnosed cohort, making them suitable for clinical screening application.

## Materials and methods

### Patients and sample collection

Patients with clinically diagnosed AD were recruited from the outpatient and inpatient departments of Geriatric Psychiatry, Affiliated Kangning Hospital of Ningbo University. HC individuals were recruited from healthy volunteers who agreed to participate in clinical research. All participants signed written informed consent. The study followed the research protocol approved by the Clinical Research Ethics Committee of Affiliated Kangning Hospital of Ningbo University (approval number: NBKNYY-2023-LC-51). The study was registered in the Chinese Clinical Trial Registry (registration number: ChiCTR2300075854).

The clinical team consisted of one associate chief psychiatrist, one attending physician, and two neuropsychological assessors, all of whom received standardized training. Subjects enrolled in the AD group met the NIA-AA 2011 clinical diagnostic criteria for AD (8).

A total of 211 participants’ serum samples were collected, including 115 clinically diagnosed AD patients. Using the Clinical Dementia Rating (CDR) scale, these patients were divided into mild AD (CDR = 1, n=26), moderate AD (CDR = 2, n=39), and severe AD (CDR = 3, n=50). The HC group comprised 96 individuals. General information was recorded, and neuropsychological assessment questionnaires were administered. All participants underwent brain MRI scanning, including axial and coronal views. For AD patients, the medial temporal lobe atrophy (MTA) scale was required to be: for age <75 years, grade 2 or higher; for age ≥75 years, grade 3 or higher. All subjects had normal liver and kidney function within one month prior to serum sampling. Subjects with a history of long-term chronic medication or any drug use within one month before blood collection were excluded. In the participant recruitment phase, we attempted to roughly match AD patients and healthy controls for general habitual dietary patterns, including major sources of dietary copper such as seafood and organ-meat consumption, based on routine clinical inquiry. Formal validated food-frequency questionnaires for quantitative dietary-copper assessment were not performed in the present study.

### Serum tot-Cu detection

Tot-Cu was detected using atomic absorption spectrometry for copper. Reagents: (1) 65%–68% guaranteed reagent nitric acid (Sinopharm Group), concentrated hydrochloric acid (Sinopharm Group), concentrated sulfuric acid (Sinopharm Group); (2) ultrapure water (Dura series ultrapure water system); (3) copper standard solution (National Nonferrous Metals and Electronic Materials Analysis and Testing Center, 500 mg/mL GBW(E)080122 copper element). Sample extraction: Samples were diluted 1:9 with tritonX-100, acidified with 2M HCl, and ashed with a mixture of 20 mL nitric acid, perchloric acid, and sulfuric acid (ratio 10:6:4). Standard configuration: standards were prepared according to the standard solution concentration table. Sample loading volume was 100 µL (iCE™ 3400 AAS atomic absorption spectrometer). The absorbance values of standards were used to fit an equation, and sample concentrations were calculated by substituting sample absorbance into the equation.

### Serum free-Cu detection

We innovatively used ultrafiltration to detect the key indicator serum free-Cu. To select the appropriate ultrafiltration membrane, we conducted a preliminary experiment; the process and data are shown in **Supplementary Table S1**. Detection process: First, serum samples were ultrafiltered using a 10K membrane (Millipore), allowing free-Cu to completely pass through while protein-bound copper was fully retained. The ultrafiltrate was then used to detect free-Cu by atomic absorption spectrometry, following the same procedure as for tot-Cu detection.

### Serum iCp and eCp detection

Human ceruloplasmin (CP/CER) ELISA kit was used. Detection method: Standards and samples were added to microplates pre-coated with anti-ceruloplasmin antibodies, followed by HRP-labeled detection antibodies. After incubation and thorough washing, the substrate TMB was added. TMB is converted to blue color under peroxidase catalysis and turns yellow upon addition of acid. The intensity of color is positively correlated with ceruloplasmin (CP/CER) concentration in the sample. Absorbance (OD value) was measured at 450 nm using a microplate reader, and sample activity was calculated.

### Data analysis

All statistical analyses were performed using SPSS Statistics 29.0.1, and graphs were generated using Prism 9.0 and SPSS. Continuous variables were described as mean ± standard deviation, and categorical variables were described as counts and percentages. Group comparisons for categorical variables were performed using the chi-square (χ²) test. For continuous variables, the Kolmogorov-Smirnov test was first used to assess the overall normality of the data distribution. For non-normally distributed continuous variables, nonparametric tests were used for group comparisons: the Mann-Whitney U test was used for comparisons between two groups, and the Kruskal-Wallis H test was used for comparisons among three groups. For normally distributed continuous variables, the independent samples t-test was used for comparisons between two groups, and analysis of variance (ANOVA) was used for comparisons among three groups. A P value < 0.05 was considered statistically significant for all analyses. Receiver operating characteristic (ROC) curves were used to describe the diagnostic performance of clinical variables, and the area under the curve (AUC) was used to quantify diagnostic efficacy.

## Results

### Comparison of general information between AD group and healthy control group

The study included 211 participants: 115 AD patients (39 males, 76 females) and 96 HC (42 males, 54 females). The mean age was 72.08 ± 7.09 years in the AD group and 71.39 ± 7.06 years in the HC group. Mini-Mental State Examination (MMSE) scores were 9.60 ± 4.32 in the AD group and 24.07 ± 2.30 in the HC group. Comparisons between the two groups for variables including sex, age, education level, MMSE total score, and eight sub-scores (orientation, immediate memory, calculation, recall, comprehension/naming, execution, writing, copying) showed significant differences only in MMSE scores (**Table 1**).

**Table 1:** Comparison of general information between the AD group and the HC group.

Clinical data	AD(n=115)	HC(n=96)	Z/χ^2^	P
Age	72.08±7.09	71.39±7.06	-0.567	0.571
Gender			2.141	0.143
Male	39(33.91%)	42(43.75%)		
Female	76(66.09%)	54(56.25%)		
Education level			0.875	0.646
Illiterate	36(31.30%)	26(27.08%)		
Primary school	30(26.09%)	23(23.96%)		
Middle school and above	49(42.61%)	47(48.96%)		
Course of disease	2.54±1.70	N/A		
MMSE Scores	9.60±4.32	24.07±2.30	-12.514	**<0.001**
Orientation	3.81±2.51	8.31±1.16	-11.222	**<0.001**
Instantaneous Memory	0.96±0.43	2.21±0.83	-10.385	**<0.001**
Calculate	0.06±0.27	4.08±0.84	-13.548	**<0.001**
Memory	0.08±0.33	2.47±0.50	-13.509	**<0.001**
Retell-Understand-Name	1.20±0.83	2.99±0.85	-10.872	**<0.001**
Execute	2.50±0.99	2.52±0.50	-2.314	**0.021**
Write	0.83±0.38	0.96±0.20	-3.006	**0.003**
Copy	0.17±0.38	0.53±0.50	-5.457	**<0.001**

Statistical analysis: Group comparisons for categorical variables were performed using the chi-square (χ²) test; group comparisons for continuous variables were performed using the Mann-Whitney U test, and P values are indicated.

Bold values indicate statistically significant comparisons where *P* < 0.05.

### Comparison of general information among AD subgroups

We also compared information among AD patients of different severities: mild AD (4 males, 22 females), moderate AD (17 males, 22 females), severe AD (18 males, 32 females). The mean ages were 72.73 ± 6.93 years (mild), 71.46 ± 7.32 years (moderate), and 72.22 ± 7.10 years (severe). MMSE scores were 13.23 ± 1.86 (mild), 12.28 ± 2.47 (moderate), and 5.62 ± 2.84 (severe). Comparisons among the three groups for sex, age, education level, MMSE total score, and eight sub-scores showed significant differences only in MMSE total score and some sub-scores (**Table 2**).

**Table 2:** Comparison of general clinical data among different AD patient groups.

Clinical data	Mild AD (n=26)	Moderate AD (n=39)	Severe AD (n=50)	H/χ^2^	P
Age	72.73±6.93	71.46±7.32	72.22±7.10	0.571	0.752
Gender				5.709	0.058
Male	4 (15.38%)	17 (43.59%)	18 (36.00%)		
Female	22 (84.62%)	22 (56.41%)	32 (64.00%)		
Education level				2.840	0.585
Illiterate	10 (38.46%)	11 (28.21%)	15 (30.00%)		
Primary school	6 (23.08%)	8 (20.51%)	16 (32.00%)		
Middle school and above	10 (38.46%)	20 (51.28%)	19 (38.00%)		
Course of disease	2.29±1.43	2.43±1.71	2.76±1.82	1.057	0.590
MMSE Scores	13.23±1.86	12.28±2.47	5.62±2.84	81.572	**<0.001**
Orientation	5.77±1.42	5.36±2.02	1.58±1.20	74.499	**<0.001**
Instantaneous Memory	1.23±0.59	0.95±0.22	0.82±0.39	14.643	**<0.001**
Calculate	0.12±0.43	0.05±0.22	0.04±0.20	0.513	0.774
Instantaneous Memory	0.15±0.46	0.08±0.35	0.04±0.20	1.812	0.404
Calculate	1.69±0.55	1.72±0.51	0.54±0.68	55.868	**<0.001**
Memory	2.92±0.27	2.95±0.22	1.92±1.26	30.648	**<0.001**
Retell-Understand-Name	0.92±0.27	0.97±0.16	0.66±0.48	17.120	**<0.001**
Execute	0.42±0.50	0.21±0.41	0.02±0.14	19.573	**<0.001**

Statistical analysis: Group comparisons for categorical variables were performed using the chi-square (χ²) test; group comparisons for continuous variables were performed using the Kruskal-Wallis H test, and P values are indicated.

Bold values indicate statistically significant comparisons where *P* < 0.05.

### Comparison of peripheral serum copper metabolite levels between AD patients of different severities and HC

Significant differences were found in eCp, iCp, eCp/iCp, tot-Cu, and free-Cu levels among the mild AD, moderate AD, severe AD, and HC groups (P<0.05) (**Table 3**).

**Table 3:** Comparison of peripheral serum copper metabolite levels among the mild, moderate, and severe AD groups and the HC group.

Peripheral serum copper metabolite	Mild AD n=26	Moderate AD n=39	Severe AD n=50	HC n=96	H	P
eCp	238.302802±15.058226	241.144992±14.331394	239.602111±15.814745	322.876496±15.137375	156.463	<0.001
iCp	534.620818±28.097363	533.349408±25.956771	538.011547±24.968710	682.509122±25.862872	156.487	<0.001
eCp/iCp	0.446969±0.037074	0.452945±0.031614	0.446619±0.040101	0.473704±0.027804	24.622	<0.001
tot-Cu	1.386633±0.080417	1.400059±0.077496	1.380638±0.071970	0.973085±0.075141	156.636	<0.001
free-Cu	0.037873±0.004618	0.037731±0.0036918	0.038192±0.0037812	0.02077604±0.003305669	156.318	<0.001

Statistical analysis was performed using the Kruskal-Wallis H test, and H values and P values are indicated.

We further compared the levels of five serum copper metabolites between any two groups (**Figure 1**). The results showed no statistically significant differences in any serum copper metabolite levels between mild, moderate, and severe AD groups (P>0.05). However, post-hoc power analyses revealed that both the overall omnibus comparison and all pairwise comparisons across AD severity subgroups exhibited statistical power below 80 % (**Supplementary Table S3**). These negative findings should therefore be interpreted with caution. eCp, iCp, and eCp/iCp levels in mild, moderate, and severe AD groups were all lower than those in the HC group (P<0.01). tot-Cu and free-Cu levels in mild, moderate, and severe AD groups were all higher than those in the HC group (P<0.001).

**Figure 1:**
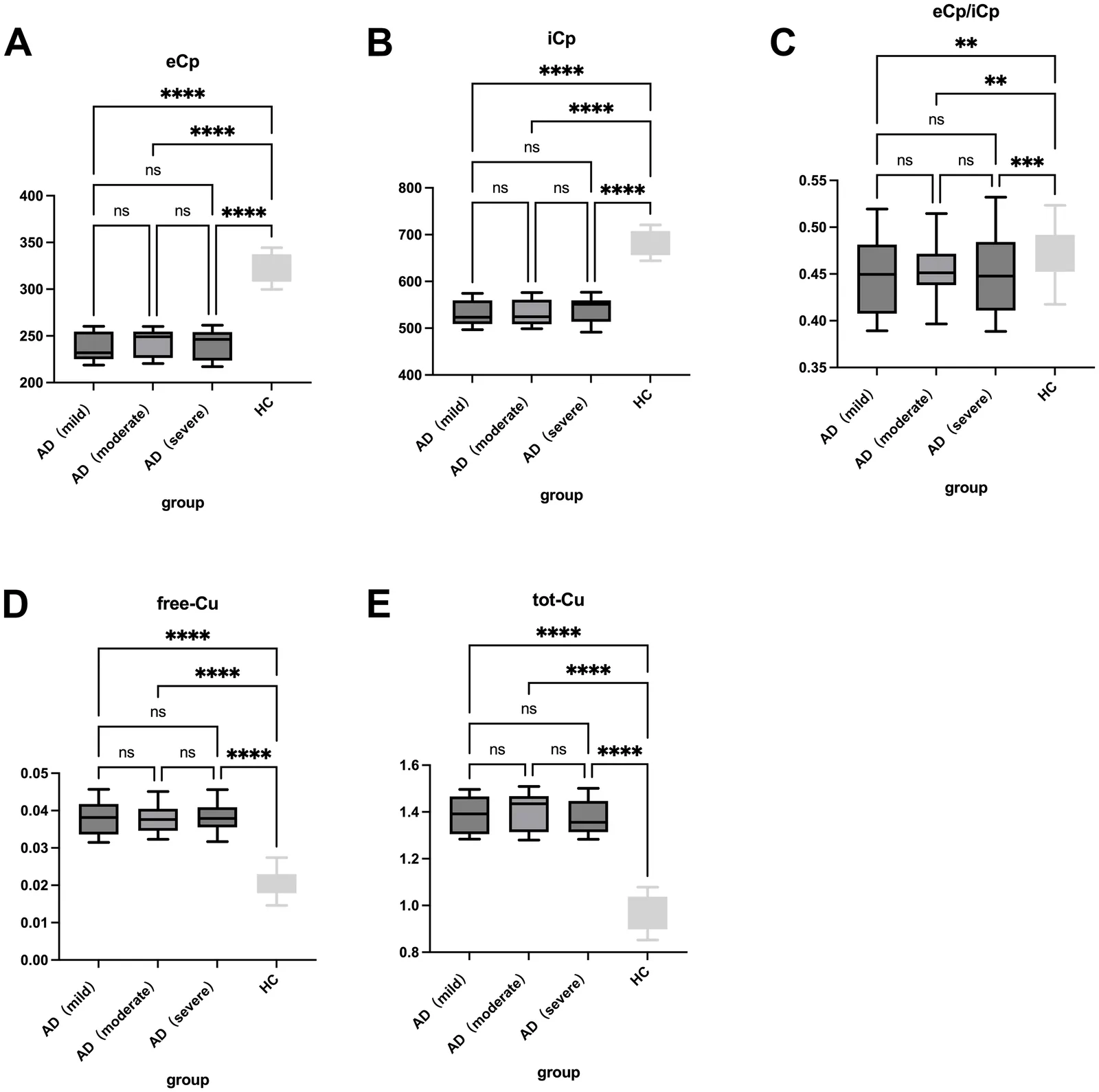
Pairwise comparisons of peripheral serum copper levels among mild, moderate, and severe AD patients and the HC group. **(A)** Pairwise comparisons of ceruloplasmin enzyme activity **(eCp)** between different groups; **(B)** Pairwise comparisons of immunological ceruloplasmin concentration **(iCp)** between different groups; **(C)** Pairwise comparisons of the Cp ratio (eCp/iCp) between different groups; **(D)** Pairwise comparisons of free copper **(free-Cu)** between different groups; **(E)** Pairwise comparisons of serum total copper concentration **(tot-Cu)** between different groups. Statistical analyses were performed using the independent samples t-test or the Mann-Whitney U test. **(A-E)** **p < 0.01, ***p < 0.001, ****p < 0.0001.

### Diagnostic performance of serum copper metabolites for AD

ROC curves comparing all clinical variables, including serum copper metabolites, against disease status were generated to evaluate diagnostic efficacy (**Figure 2**, **Supplementary Table S2**).

**Figure 2:**
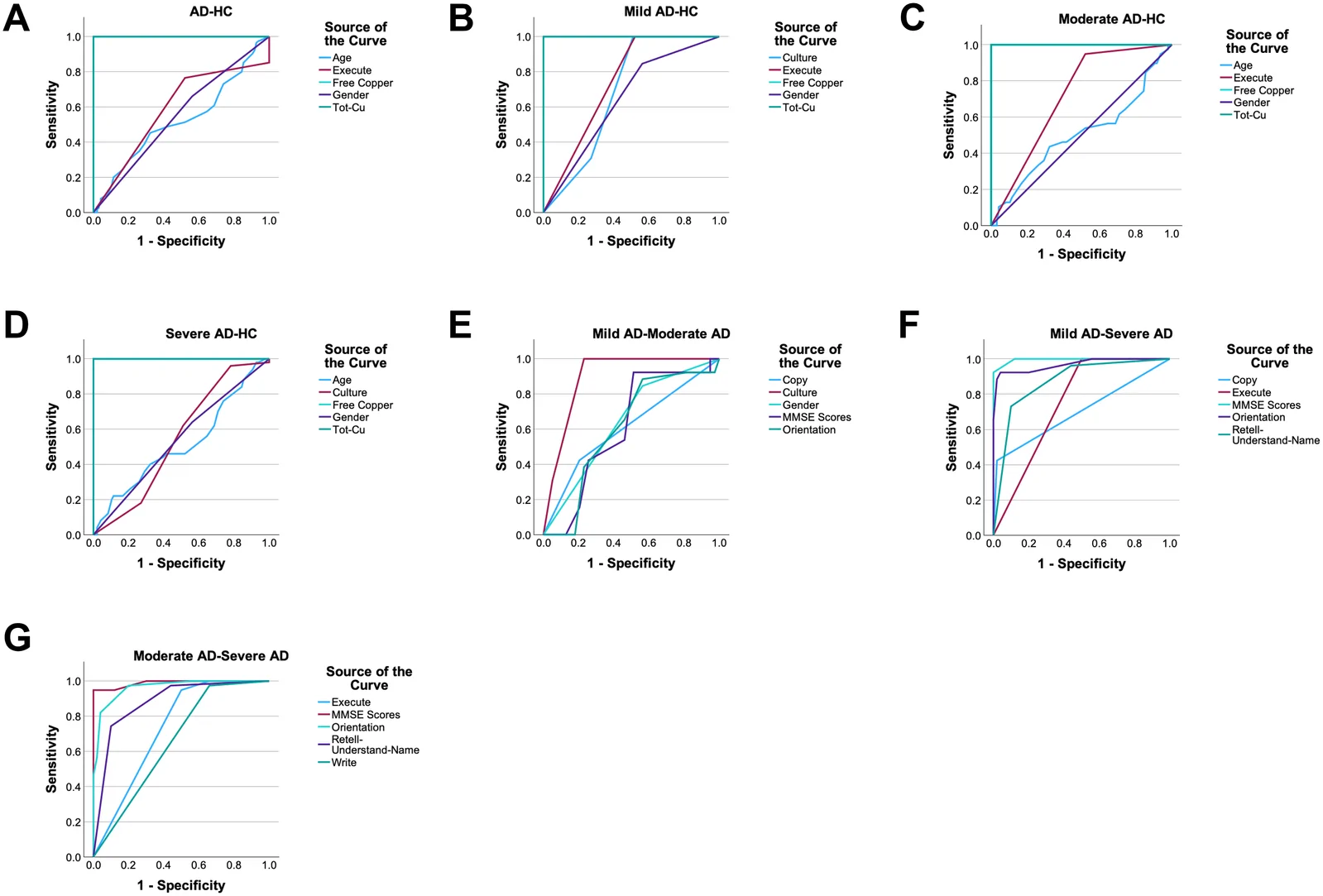
ROC curves of diagnostic performance between AD groups of different severities and the HC group. **(A)** ROC curves of diagnostic performance between the AD group and the HC group; **(B–D)** ROC curves of diagnostic performance between the mild, moderate, and severe AD groups and the HC group, respectively; **(E–G)** ROC curves of diagnostic performance for pairwise comparisons among the mild, moderate, and severe AD groups.

The results showed that when distinguishing AD of any severity from HC, free-Cu and tot-Cu levels achieved the highest diagnostic efficacy, superior to the MMSE scale. When comparing between different severity AD groups, the five serum copper metabolites showed no significant diagnostic advantage over other clinical variables; rather, MMSE total score or education level had higher AUC values. In the mild AD vs. moderate AD ROC curve, education had the highest AUC (0.894). In the mild/moderate AD vs. severe AD ROC curves, MMSE total score had the highest AUC (0.995 and 0.989, respectively).

## Discussion

Copper metabolites in human serum may participate in the pathological process of AD and exhibit heterogeneity in AD patients. Our study further validated this view and comprehensively analyzed five human serum copper metabolites. We found that levels of the five copper metabolites (eCp, iCp, eCp/iCp, tot-Cu, free-Cu) in AD patients were significantly different from those in HC, but no differences were found among AD patients of different severities. Among them, free-Cu and tot-Cu showed excellent diagnostic performance for AD. For free-Cu detection methodology, we used ultrafiltration instead of the traditional Walshe formula and personalized pre-experimentation for membrane selection.

### Ultrafiltration for free-Cu detection provides more accurate and objective data

This study used ultrafiltration combined with atomic absorption spectrometry to detect serum free-Cu. This method (26) is more accurate and objective than the traditional Walshe formula calculation. The Walshe formula [bound copper (µmol/L) = serum ceruloplasmin (mg/L) × (0.3/100) × (1000/63.6); free copper = total copper – bound copper] is highly dependent on the accuracy of Cp measurement and is based on idealized assumptions (i.e., copper in the human body is completely bound to Cp and reaches saturation). In reality, the Cp binding ratio is certainly not 100% (21). Errors in Cp detection can easily lead to large deviations in free-Cu results, sometimes even producing negative values. Ultrafiltration directly measures free copper through physical filtration, avoiding the shortcomings of the Walshe formula. Our experiments further verify the limitations of the Walshe formula (**Figure 3**). As illustrated in this bar-and-scatter plot, free-Cu measured by the ultrafiltration method yields exclusively positive values across all participants, whereas free-Cu calculated by the Walshe formula frequently produces physically implausible negative values. The main source of this error may be that the assumption that bound copper accounts for 0.3% of ceruloplasmin is overly absolute and fails to account for inter-individual variability. Preliminary experimental results of this study suggest that the 10 K ultrafiltration membrane can almost completely retain copper-albumin complexes in the serum samples. The albumin concentration in the ultrafiltrate measured by the bromocresol green albumin assay kit was below the lower limit (0.1 mg/mL), confirming that virtually no copper-albumin complexes existed in the filtrate obtained with the 10 K ultrafiltration membrane. Accordingly, the measured free-Cu values are accurate. The average proportion of free-copper in the ultrafiltrate was determined to be 2.53%, which is consistent with the ranges reported in the literature (26). In addition to ultrafiltration, another fluorescent probe detection method (27) for free-Cu is also more efficient than the Walshe formula method. However, that method is susceptible to interference from metals such as Fe, Zn, Mg, Ca, and from the fluorescence absorption spectra of hemoglobin and albumin. Therefore, the ultrafiltration method used in this experiment has greater specificity and reliability, providing methodological advantages for free-Cu detection.

**Figure 3:**
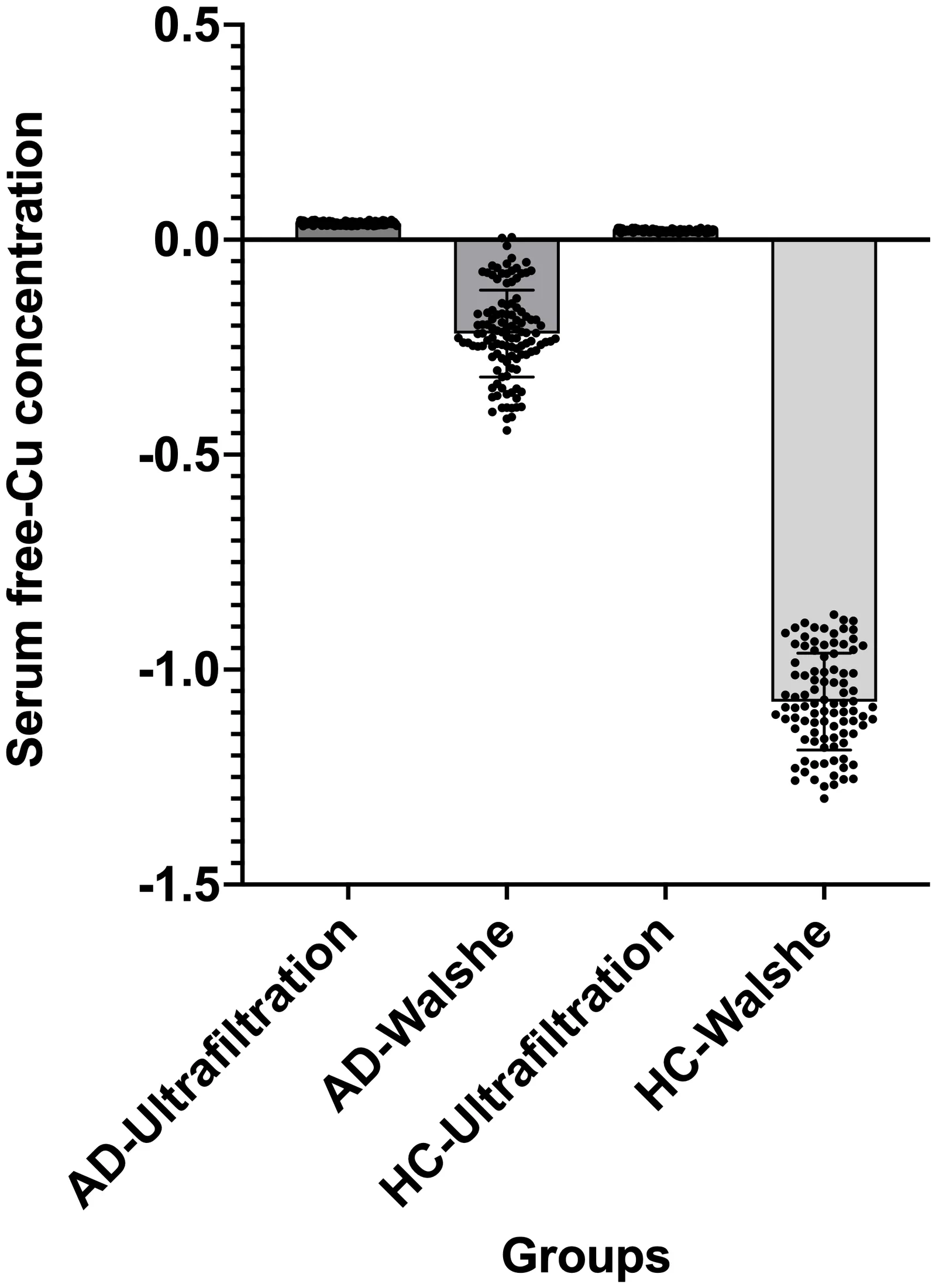
Comparison of serum free-Cu values measured by ultrafiltration versus calculated by the Walshe formula in HC and AD participants. Bar-and-scatter plot displaying group mean ± standard deviation (bar) overlaid with individual participant data points (scatter). Free-Cu obtained by ultrafiltration yields only positive values, whereas the Walshe formula frequently generates non-physiological negative free-Cu estimates in both HC and AD cohorts.

### Abnormal peripheral serum copper metabolite levels in clinically diagnosed AD patients

Our results showed that peripheral serum copper metabolite levels in the mild, moderate, and severe clinically diagnosed AD groups were significantly different from those in the HC group, while no differences were found among the AD subgroups. Specifically, eCp, iCp, and eCp/iCp levels in the HC group were significantly higher than those in the mild, moderate, and severe AD groups, whereas tot-Cu and free-Cu levels were significantly lower than those in the three AD groups.

It should be noted that dietary copper intake represents a plausible confounding factor for circulating serum copper measurements. Although general habitual dietary patterns were roughly balanced between groups according to clinical inquiry, without quantitative food-frequency questionnaire data, we cannot definitively exclude residual dietary contributions to the elevated tot-Cu observed among AD patients. Accordingly, the higher tot-Cu in the AD cohort may reflect a composite of AD-related copper metabolic dysregulation and potential unmeasured dietary influences.

### Tot-Cu

In this study, tot-Cu levels in all clinically diagnosed AD groups were significantly higher than in HC, consistent with previous findings (18, 28). A meta-analysis including 2929 AD patients and 3547 healthy controls (29) showed increased tot-Cu in AD patients, but heterogeneity was high (I² = 95.9%), indicating the need for more samples. Another study (29) found a positive correlation between peripheral serum tot-Cu level and AD incidence: for each 1 µmol/L increase in tot-Cu, the probability of developing AD increased fourfold.

Elevated tot-Cu exacerbates AD pathology and interacts with cerebral Aβ aggregation, correlating negatively with CSF Aβ1-42 (30). Increased peripheral tot-Cu initiates neuroinflammation, impairs Aβ clearance, suppresses microglial activity, and drives a self-amplifying pathological cycle (31). Copper binds to the amyloid precursor protein to modulate the Aβ cascade; higher peripheral tot-Cu facilitates copper accumulation within Aβ plaques and neurofibrillary tangles and alters APP expression (32). Copper-Aβ redox cycling generates H_2_O_2_, promoting Aβ-oligomer formation and lipid peroxidation (33). At glutamatergic synapses, dysregulated copper interacts with redox-active Aβ to produce neurotoxic soluble Aβ, aggravating Aβ deposition (34).

### Free-Cu

Free-Cu is the free form of copper in the peripheral circulation. With a small molecular weight, it readily crosses the BBB and directly or indirectly affects the CNS. When its concentration in the brain exceeds 1.6 µmol/L, it becomes neurotoxic.

Free-Cu has a clear effect on AD progression: each 1 µmol/L increase raises the probability of developing AD threefold (29). Studies in AD animal models have shown that ingesting a small amount of copper (approximately 0.13 mg/L) via drinking water can double central nervous system free-Cu levels, reduce intracranial Aβ clearance (35), increase hippocampal copper levels, increase Aβ deposition, enhance oxidative stress responses, and lead to significant behavioral and cognitive deficits (36).

Our study showed that peripheral free-Cu levels in clinically diagnosed AD patients of all severities were higher than in HC. A large-sample meta-analysis (29) was consistent with our findings, but the study noted that the standardized mean difference for free-Cu was very low and heterogeneity was high (I²=91.5%), and some studies even reported negative free-Cu values, presumably due to inappropriate detection methods, requiring further validation. Our use of ultrafiltration combined with atomic absorption spectrometry to accurately measure free-Cu values makes the results more objective and scientific.

The origins of elevated serum free-Cu in AD remain incompletely clarified. Genetic evidence links ATP7B variants (rs1061472, rs732774) to higher serum free-Cu and elevated AD risk (29). A location-dependent copper-shift model proposes that free-Cu acts as the central toxic effector: copper redistributes from tightly-bound functional pools toward loosely-bound toxic fractions, disrupting peripheral and brain copper buffering and triggering mitochondrial dysfunction, oxidative stress, inflammation and tau hyperphosphorylation (37). Elevated free-Cu also aggravates AD-related oxidative stress; cortical free-Cu correlates with oxidative injury, and free-Cu-Aβ interaction catalyzes reactive-oxygen-species-mediated neurotoxicity via hydroxyl-radical-generating redox cycles (38).

### ICp, eCp, eCp/iCp

This study found that peripheral eCp and iCp levels in the clinically diagnosed AD group were lower than those in the healthy control group. The findings regarding iCp changes in AD are inconsistent across studies. Some studies report results similar to ours (39); however, others have found no significant difference between groups (20). A large-sample meta-analysis showed no statistical difference in iCp levels between the two groups (29). In contrast, fewer studies have focused on eCp changes in AD. The limited available evidence supports our observed trend, showing low eCp expression in AD patients (40). Another meta-analysis of 19 studies included only three that measured eCp levels, and their results were consistent with ours (41).

Given that using iCp or eCp alone is controversial and may be influenced by multiple factors, their ratio may have greater clinical significance. This approach draws on experience from WD diagnosis. In WD, ATP7B gene mutations prevent copper from being properly incorporated into newly synthesized ceruloplasmin, producing inactive apoceruloplasmin, which is rapidly degraded upon secretion into the blood (42, 43). Routine immunological measurement of iCp concentration cannot distinguish between active holoceruloplasmin and inactive apoceruloplasmin, potentially leading to missed diagnoses (42, 44). If AD involves a similar copper metabolism disorder, a scenario of “normal iCp concentration but reduced eCp activity” may also occur.

Therefore, to more accurately assess ceruloplasmin status in AD, we introduced the eCp/iCp ratio as a core analytical variable. This ratio is not affected by factors such as age, sex, or medication use and has been used to evaluate copper metabolism abnormalities in various diseases. Its stability is superior to iCp alone, which is susceptible to confounding factors (45, 46). Based on previous meta-analyses suggesting an association between oxidative stress and ceruloplasmin (41), this study focused on the relationship between the eCp/iCp ratio and AD risk.

Our study found that the eCp/iCp ratio in the AD group was significantly lower than in the HC group, indicating a relative deficiency of holoceruloplasmin (the fully active form) in AD patients. This suggests that ceruloplasmin biosynthesis may be impaired in AD patients, resulting in defective copper incorporation (or premature loss) and a shift from active holoceruloplasmin to inactive apoceruloplasmin, i.e., a decreased eCp/iCp ratio. This finding is consistent with previous studies showing elevated serum apo-Cp (functionally deficient Cp) levels in AD patients (47, 48). Some studies suggest this phenomenon may be related to free-Cu interference with hepatocytes and decreased liver function (49). Moreover, a low eCp/iCp ratio indicates the presence of a “defective” ceruloplasmin, whereby the concentration measured immunologically is higher than that expected from its enzymatic activity, similar to observations in WD, suggesting that not all ATP7B gene mutations reduce iCp concentration; some late-onset cases may have normal iCp values that mask underlying copper imbalance (45). In addition to synthesis defects, oxidative damage to ceruloplasmin and post-translational modifications may also restrict its activity (50). In summary, the eCp/iCp ratio objectively reflects the true status of functional ceruloplasmin in the body and has become an important indicator for monitoring copper metabolism abnormalities in WD and AD, as well as for evaluating the efficacy of copper chelation therapy.

### Peripheral copper metabolic dysregulation may contribute to the onset of AD

Our results revealed significant differences in peripheral serum copper metabolites between AD patients and healthy controls (HC), whereas no inter-group differences were observed among mild, moderate, and severe AD subgroups. We hypothesize two potential roles for peripheral copper dyshomeostasis in AD progression: first, that peripheral copper homeostatic disturbance represents an early initiating event of AD pathology; second, that it acts as a progression-driving factor that continuously aggravates dementia. Our findings lend more support to the former hypothesis.

According to the first hypothesis, abnormalities in peripheral serum copper homeostasis emerge as early as the predementia and mild cognitive impairment (MCI) stages. After the disease progresses to the dementia phase, self-amplifying pathological loops formed by cerebral Aβ-tau-oxidative-stress cascades can independently drive disease deterioration, and peripheral circulating copper biomarkers no longer change gradually alongside worsening cognitive impairment. This interpretation is consistent with our cross-sectional observations.

The pathological process proceeds through several successive stages. At the MCI stage, elevated serum free-Cu serves as an initial pathological trigger. Increased free-Cu is capable of crossing the blood-brain barrier (51). Once entering the central nervous system, it binds to Aβ peptides, catalyzes reactive-oxygen-species production, facilitates Aβ oligomerization, exacerbates tau hyperphosphorylation, and initiates oxidative-stress-mediated injury, laying the groundwork for subsequent AD pathology (52). Clinical studies have demonstrated that free-Cu levels are already higher in amnestic MCI individuals without overt dementia than in healthy elderly subjects (53).

As pathology advances, self-sustaining pathological loops are established within the brain, and further disease progression becomes independent of additional elevations in peripheral copper. Once Aβ plaques and tau neurofibrillary tangles form in the brain, local positive-feedback pathology is initiated: plaques sequester cerebral copper ions, and accumulated local copper further augments Aβ aggregation, oxidative stress, and microglial activation (54). Neuroinflammation in turn promotes additional Aβ and tau pathology, forming a closed loop that perpetuates neurodegeneration (54). That is, after dementia sets in, cerebral pathological loops that have already been triggered can continue to drive progressive cognitive decline even if serum free-Cu and tot-Cu do not rise further (51). This partly explains why patients with more severe dementia in our cohort did not exhibit further elevations in peripheral copper markers (52).

Meanwhile, decoupling between peripheral and central copper occurs (52). Under AD pathological conditions, cerebral copper undergoes redistribution (51). Large amounts of copper are sequestered within Aβ senile plaques, and local brain-parenchyma copper homeostasis continues to deteriorate (54). Nevertheless, serum copper concentrations do not increase in parallel with advancing cerebral pathology, resulting in dissociation between peripheral biomarkers and genuine central pathological status (55).

By contrast, the second hypothesis regards peripheral copper metabolic disturbance as a driver of disease progression. This view predicts that peripheral copper biomarkers should deteriorate progressively as dementia severity increases. A small number of cross-sectional studies appear to support this idea, though these works possess notable limitations. One clinical study reported a negative correlation between serum tot-Cu and cognitive scores in AD patients, with higher tot-Cu corresponding to more severe dementia (56). Another investigation calculated free-Cu via regression models and found this parameter negatively correlated with MMSE scores among AD patients, implying greater cognitive impairment in subjects with higher peripheral copper levels (30). However, both studies were cross-sectional without longitudinal dynamic data; they only assessed either tot-Cu or computationally derived free-Cu, lacking comprehensive profiling of multiple serum copper-related indices.

Collectively, our cross-sectional data are more consistent with the notion that copper dyshomeostasis functions predominantly as an early initiating event in AD. Pronounced alterations in peripheral copper biomarkers arise early in AD; after dementia develops, peripheral copper markers show no graded changes despite progressive clinical severity, and subsequent disease progression is mainly propelled by intrinsic cerebral pathological loops. Nevertheless, this remains a hypothesis derived from observational data rather than definitive conclusions. To discriminate between these two competing interpretations, longitudinal cohorts starting from MCI or preclinical AD are required to track dynamic changes in copper biomarkers across the disease course. Combined measurements of cerebrospinal fluid markers or cerebral metal imaging will help compare copper trajectories in the periphery versus the central nervous system.

### Diagnostic performance of peripheral serum copper metabolites for clinically diagnosed AD

This study evaluated the ability of peripheral serum copper metabolites and other clinical indicators to discriminate between clinically diagnosed AD patients of different severities and HC, and attempted to further differentiate AD severity. Diagnostic performance was expressed using the area under the receiver operating characteristic curve (AUC). The results showed that when distinguishing these AD patients from HC, or when differentiating AD of any severity from HC, the indicators with the highest diagnostic efficacy were tot-Cu and free-Cu levels (**Figures 2A–D**; **Supplementary Table S2**). These findings suggest that tot-Cu and free-Cu can effectively discriminate patients with clinically phenotyped AD from HC, with diagnostic efficacy superior to the MMSE scale, suggesting that serum copper metabolites may serve as objective biomarkers for clinical screening of clinically-diagnosed AD. However, when distinguishing between clinically diagnosed AD patients of different severities, none of the five peripheral serum copper metabolites showed significant diagnostic advantages; instead, MMSE total score demonstrated greater advantage in this regard (**Figures 2E–G**, **Supplementary Table S2**).

Previous studies (39) have suggested that peripheral free-Cu and tot-Cu levels can distinguish AD from HC, and that the antioxidant capacity of AD patients is significantly lower than that of HC, indicating a more prominent role of oxidative stress in AD, which is closely related to copper; this may partly explain the differences in serum copper levels between the two groups.

Currently, studies on differences in peripheral serum copper levels among AD patients of different severities are scarce. Our results show that neither tot-Cu, free-Cu, nor the functional eCp/iCp ratio can effectively distinguish between different severities of clinically diagnosed AD. Nevertheless, post-hoc power analyses indicated insufficient statistical power for these severity-stratified subgroup comparisons (**Supplementary Table S3**); accordingly, we cannot fully exclude potential type-II error, and these null findings should not be exclusively attributed to biological decoupling between copper metabolism and AD severity. This observation may also be related to individual differences in the distribution and impact of various pathogenic factors during AD development and progression. First, some studies (57) have shown that CSF Aβ42 levels, Aβ-PET plaque uptake values, total tau (tot-tau), and phosphorylated tau (p-tau181) in AD patients are not correlated with MMSE scores, suggesting that pathological biomarker levels may not be directly related to clinical severity in AD. Second, although oxidative stress is an important pathogenic mechanism in AD (58), and copper (especially free-Cu, which can freely cross the BBB) can affect oxidative stress levels in the brain, a study using the antioxidant lipoic acid to treat AD patients of different severities (59) found that the treatment was ineffective for moderate AD patients and only partially effective for mild AD patients, and improvements in the Alzheimer’s Disease Assessment Scale (ADAS) and MMSE scores were inconsistent. This indicates that there may also be no clear linear relationship between oxidative stress levels and disease severity in AD patients.

In summary, although peripheral serum copper metabolism-related indicators may be involved in processes such as Aβ deposition, oxidative stress, and tau pathology, the interplay of multiple pathogenic factors in AD patients of different severities, together with limited statistical power for subgroup contrasts, means we cannot draw firm conclusions regarding whether copper markers can differentiate AD severity.

## Limitations

This study has several limitations. First, the sample size is still insufficient. Although 211 subjects were enrolled, as a cross-sectional study, the contribution of sample size to the results is not convincing. In the future, we will further deepen the research and develop cohort studies to observe the dynamic changes between disease progression and serum copper levels. Second, the AD patients in this study were enrolled solely based on the NIA-AA 2011 clinical diagnostic criteria, without biomarker confirmation of amyloid-β or tau pathology. Therefore, biologically-confirmed AD cannot be strictly differentiated from other types of dementia, and potential inclusion of non-AD dementia cases may introduce analytical bias. Notably, our overall AD cohort had a mean MMSE score of 9.6, with most patients categorized as CDR 2-3 (moderate-to-late-stage dementia). Although our cohort contained a small subgroup of mild AD (CDR = 1, n=26), the study population is dominated by moderate-to-late-stage cases. Therefore, our findings cannot be generalized to true early-stage preclinical or prodromal AD, and the generalizability of conclusions should be interpreted with caution. Third, patients with mild cognitive impairment (MCI) were not included. This study has already found that serum copper levels are significantly abnormal in early AD. In future studies, we will enroll MCI patients and compare their serum copper levels with those of AD patients. Fourth, sample sizes were unbalanced across AD severity subgroups, with a relatively small number of patients in the mild AD group (n=26). Post-hoc power analyses demonstrated that statistical power for both omnibus and pairwise subgroup comparisons was below 80 % (**Supplementary Table S3**). Potential type-II error cannot be excluded for the null between-subgroup findings; these non-significant results should not be interpreted as definitive proof that copper metabolites bear no biological relation to AD severity. Replication in larger cohorts with balanced subgroup sizes, especially with an expanded mild AD sample, is urgently needed. Fifth, quantitative data on dietary copper intake were not collected in the present study. Although we attempted to roughly match general habitual dietary patterns (including seafood and organ-meat consumption, major dietary copper sources) between AD patients and healthy controls via clinical inquiry at recruitment, formal validated food-frequency questionnaires for quantitative copper-intake evaluation were absent. Dietary copper intake constitutes an important potential confounder for circulating copper biomarkers. Therefore, the observed inter-group differences in serum tot-Cu and free-Cu cannot be fully disentangled from possible residual dietary influences; part of the group difference could theoretically originate from dietary intake rather than AD-related pathological changes. Further well designed studies incorporating detailed, validated dietary assessment are warranted to clarify this issue.

## Conclusion

This study adopted the ultrafiltration method to accurately and objectively measure peripheral serum copper-related levels in subjects with clinically diagnosed AD. Compared with healthy controls, AD patients exhibited decreased levels of eCp, iCp, and eCp/iCp, alongside significantly elevated free-Cu and tot-Cu. Nevertheless, no statistically significant differences were observed for any of these indicators across subgroups of clinically phenotyped AD with varying severity. This study confirms prominent peripheral copper metabolic dysregulation in patients with clinically phenotyped AD; however, due to limited statistical power in severity-stratified subgroup analyses, we cannot draw firm conclusions regarding whether copper metabolic abnormalities vary with disease severity. At the diagnostic level, free-Cu and tot-Cu showed excellent diagnostic performance in discriminating patients with clinically diagnosed AD from healthy controls, whereas the MMSE performed better for differentiating disease severity. These findings suggest that free-Cu and tot-Cu can serve as biomarkers for clinical screening of clinically phenotyped AD among the general population, with favorable discriminatory capacity even for mild cases within our clinical cohort. The present findings provide references for the clinical biological diagnosis of AD and mechanistic research on copper metabolic dysregulation. Further prospective studies are still required for external validation.

## Data Availability

The raw data supporting the conclusions of this article will be made available by the authors, without undue reservation.

## Supplementary material

Supplementary Table 1 Ultrafiltration pre-experiment for free copper.

Supplementary Table 2 Area under the ROC curve values for different groups.

Supplementary Table 3 *Post-hoc* and sensitivity power analysis for severity-stratified subgroup analyses among AD patients.
