# Supplementary material for: Peripheral serum copper metabolite levels measured by ultrafiltration aid in the clinical screening of Alzheimer’s disease
Source: Front Psychiatry. 2026 Oct 14;17:1954419. doi: 10.3389/fpsyt.2026.1954419 (PMC13713676; doi:10.3389/fpsyt.2026.1954419)
Supplement: Supplementary file 1 [file SupplementaryFile1.docx]

**Supplementary Materials**

**Supplementary Tables**

**Table.S1**

| sample | membrane | OD | tot-Cu（ug/ml） | free-Cu（ug/ml） | free-Cu% | albumin stock solution(mg/mL) | ultrafiltrate albumin(mg/mL) |
| --- | --- | --- | --- | --- | --- | --- | --- |
| AD001 | 100K | 0.2817 | 1.4843 | 0.1905 | 12.83 | 53.3149 | 21.6014 |
|  | 50K | 0.3659 |  | 0.2686 | 18.10 |  | 19.5120 |
|  | 22K | 0.3925 |  | 0.2933 | 19.76 |  | 10.1871 |
|  | 10K | 0.1208 |  | 0.0335 | 2.26 |  | N/A |
| AD002 | 100K | 0.2852 | 1.4883 | 0.1937 | 13.02 | 45.1271 | 21.5295 |
|  | 50K | 0.3807 |  | 0.2823 | 18.97 |  | 16.0518 |
|  | 22K | 0.4264 |  | 0.3247 | 21.82 |  | 8.1917 |
|  | 10K | 0.1260 |  | 0.0384 | 2.58 |  | N/A |
| AD003 | 100K | 0.2596 | 1.2965 | 0.1700 | 13.11 | 50.0552 | 26.8857 |
|  | 50K | 0.3573 |  | 0.2606 | 20.10 |  | 18.0859 |
|  | 22K | 0.3948 |  | 0.2954 | 22.79 |  | 9.4133 |
|  | 10K | 0.1220 |  | 0.0346 | 2.67 |  | N/A |
| AD004 | 100K | 0.2899 | 1.4553 | 0.1981 | 13.61 | 53.3923 | 21.1205 |
|  | 50K | 0.3815 |  | 0.2831 | 19.45 |  | 19.4567 |
|  | 22K | 0.4129 |  | 0.3122 | 21.45 |  | 9.7726 |
|  | 10K | 0.1244 |  | 0.0369 | 2.54 |  | N/A |
| AD005 | 100K | 0.2675 | 1.3308 | 0.1773 | 13.32 | 54.0994 | 23.5194 |
|  | 50K | 0.3687 |  | 0.2712 | 20.38 |  | 19.5673 |
|  | 22K | 0.4046 |  | 0.3045 | 22.88 |  | 10.2258 |
|  | 10K | 0.1218 |  | 0.0344 | 2.58 |  | N/A |

**Table S1. Ultrafiltration Pre-experiment for Free Copper.**Ultrafiltration pre-experiment: Five AD serum samples were ultrafiltered using four different types of filter papers, and then the albumin content was detected using the bromocresol green albumin detection kit. The detection process consisted of two steps: detection of albumin in the original solution and detection of albumin in the ultrafiltrate. Since free-Cu exists in the peripheral blood in a free form, it is required that no albumin be detected in the ultrafiltrate. Preliminary experimental results showed that among the four specifications of ultrafiltration membranes (100 K, 50 K, 22 K and 10 K), the 10 K ultrafiltration membrane yielded the optimal filtration performance. The average fraction of free‑copper in the ultrafiltrate was 2.53%, and the albumin level in the ultrafiltrate was below the detection limit of the kit, indicating that nearly all copper‑albumin complexes were retained. The worst filtration effect was observed with the 100K ultrafiltration filter paper, with an average albumin concentration of 22.93 mg/mL in the ultrafiltrate and an average free-Cu% of 13.18%. The filtration efficiency of the 10K ultrafiltration filter paper met the requirements for free-Cu detection.

Note: N/A indicates that the albumin value detected by the bromocresol green albumin detection kit was below the lower limit of detection (0.1 mg/mL).

**Table.S2**

| Group | Clinical characteristics | ROC curve values |
| --- | --- | --- |
| AD-HC | tot-Cu | 1.000 |
|  | free-Cu | 1.000 |
|  | Execute | 0.587 |
|  | Gender | 0.549 |
|  | Age | 0.523 |
| Mild AD-HC | tot-Cu | 1 |
|  | free-cu | 1 |
|  | Execute | 0.740 |
|  | Culture | 0.688 |
|  | Gender | 0.642 |
| Moderate AD-HC | tot-Cu | 1 |
|  | free-cu | 1 |
|  | Execute | 0.714 |
|  | Age | 0.503 |
|  | Gender | 0.501 |
| Severe AD-HC | tot-Cu | 1 |
|  | free-cu | 1 |
|  | Culture | 0.546 |
|  | Gender | 0.539 |
|  | Age | 0.514 |
| Mild AD-Moderate AD | Culture | 0.894 |
|  | Gender | 0.641 |
|  | MMSE | 0.610 |
|  | copy | 0.609 |
|  | Orientation | 0.607 |
| Mild AD-Severe AD | MMSE Scores | 0.995 |
|  | Orientation | 0.967 |
|  | Retell-Understand-Name | 0.873 |
|  | Execute | 0.750 |
|  | Copy | 0.702 |
| Moderate AD-Severe AD | MMSE Scores | 0.989 |
|  | Orientation | 0.963 |
|  | Retell-Understand-Name | 0.882 |
|  | Execute | 0.733 |
|  | Copy | 0.657 |

**Table S2. Area under the ROC curve values for different groups.**

**Table.S3**

| Peripheral Serum Copper Metabolite | Omnibus ANOVA‑3 groups | | | Pair‑wise comparison | | | |
| --- | --- | --- | --- | --- | --- | --- | --- |
|  | Observed f | Required f (power=0.8) | Post‑hoc Power | Contrast | Observed d | Required d (power=0.8) | Post‑hoc Power |
| eCp | 0.070297 | 0.293363 | 0.094037 | Mild vs Mod | 0.193355 | 0.720365 | 0.116894 |
|  |  |  |  | Mild vs Sev | 0.084146 | 0.686340 | 0.063625 |
|  |  |  |  | Mod vs Sev | 0.102236 | 0.605232 | 0.076032 |
| iCp | 0.081089 | 0.293363 | 0.109589 | Mild vs Mod | 0.047005 | 0.720365 | 0.053839 |
|  |  |  |  | Mild vs Sev | 0.127571 | 0.686340 | 0.081607 |
|  |  |  |  | Mod vs Sev | 0.183062 | 0.605232 | 0.135456 |
| eCp/iCp | 0.080142 | 0.293363 | 0.108118 | Mild vs Mod | 0.173457 | 0.720365 | 0.103562 |
|  |  |  |  | Mild vs Sev | 0.009063 | 0.686340 | 0.050157 |
|  |  |  |  | Mod vs Sev | 0.175198 | 0.605232 | 0.128090 |
| tot‑Cu | 0.115067 | 0.293363 | 0.176714 | Mild vs Mod | 0.170014 | 0.720365 | 0.101412 |
|  |  |  |  | Mild vs Sev | 0.078561 | 0.686340 | 0.061865 |
|  |  |  |  | Mod vs Sev | 0.259694 | 0.605232 | 0.225076 |
| free‑Cu | 0.052295 | 0.293363 | 0.073764 | Mild vs Mod | 0.033966 | 0.720365 | 0.052002 |
|  |  |  |  | Mild vs Sev | 0.075585 | 0.686340 | 0.060978 |
|  |  |  |  | Mod vs Sev | 0.123369 | 0.605232 | 0.088125 |

**Table.S3.Post-hoc and sensitivity power analysis for severity-stratified subgroup analyses among AD patients.**Cohen’s f was calculated for omnibus one-way ANOVA across three AD severity subgroups. For pairwise comparisons, Cohen’s d was adopted as the effect-size metric. Required f and required d represent the minimal effect sizes required to achieve 80 % statistical power given the corresponding sample-size configuration. All tests were two-tailed with α = 0.05. Raw unrounded mean and SD values were used for all power-calculation procedures. Since G*Power does not natively support Kruskal-Wallis H and Mann-Whitney U tests, parametric models were used as approximations; actual statistical power of non-parametric tests would be lower, and the reported power values represent upper-bound estimates.
